# Morphological and molecular characterization of *Ektaphelenchoides pini* (Massey, 1966) Baujard, 1984 (Aphelenchoididae; Ektaphelenchinae) from Iran, with morphological and taxonomic observations on some species

**DOI:** 10.21307/jofnem-2020-052

**Published:** 2020-05-25

**Authors:** Fariba Heydari, Majid Pedram

**Affiliations:** 1 Department of Plant Pathology, Faculty of Agriculture, Tarbiat Modares University, Tehran, Iran

**Keywords:** Aphelenchoidea, Diagnostics, *Ektaphelenchoides maafiae* n. syn., *E. poinari*, *E. tonekabonensis*, *E. winteri* n. comb., rDNA sequences, Taxonomy

## Abstract

*Ektaphelenchoides pini*, the type species of the genus *Ektaphelenchoides*, was recovered from wood and bark samples of a dead broadleaf forest tree collected from the forests of Golestan province in north of Iran. The recovered population is mainly characterized by massive wide spicules of males with well-developed condylus marked by indentations at the apex and simple distal tip. It was further characterized by 756 to 947 μm long females having a cephalic region slightly separated from the rest body by a shallow depression, 20 to 23 μm stylet with wide lumen lacking conophore and knobs, excretory pore (E pore) at about one metacorpus length behind it, or 92 to 106 μm from anterior end and hemizonid just posterior to it, vagina anteriorly inclined, post uterine sac (PUS) ca 1.2 times vulval body width long, posterior body region elongate conoid, ending to a filiform tip, no functional rectum, a vestigial anus and common males with dorsally convex tail ending to an elongate spike and two pairs of precloacal (P2) and caudal (P3) large papillae at 5 to 6 μm distance anterior to cloacal opening and 30 to 41% of tail, respectively and lacking the single precloacal papilla (P1). In molecular phylogenetic analyses using small and large subunit ribosomal DNA (SSU, LSU rDNA) sequences, the Iranian population of *E. pini* fell in a clade including species of three genera *Ektaphelenchus*, *Ektaphelenchoides*, and *Devibursaphelenchus* in SSU, and a clade including species of *Ektaphelenchus* and *Ektaphelenchoides* in LSU tree, in close association with an isolate identified as *E. pini* in the latter phylogeny with high (0.99) Bayesian posterior probability (BPP). The comparisons with the type and French populations, as well as phylogenetic affinities of the species using ribosomal data, are discussed. This is the first report of *E. pini* from Iran, and its first simultaneous morphological and molecular phylogenetic study. New observations on some species of the genus were also presented and discussed.

The members of the superfamily Aphelenchoidea (Fuchs, 1937) are known to be typologically similar, primarily characterized by having a well-developed metacorpus, and some other shared characters discussed by Kanzaki and Giblin-Davis (2012), with exceptions such as the smaller metacorpus does rarely occur (Pedram et al., 2018). A wide range of feeding habit including mycetophagy, predatory, plant feeding, and insect parasitism is seen inside the superfamily (Hunt, 1993; Kanzaki and Giblin-Davis, 2012).

The biology and morphology of Ektaphelenchinae (Paramonov, 1964) members are already discussed (Hunt, 1993; Kanzaki and Giblin-Davis, 2012). Phylogenetically, the subfamily is not monophyletic, but the genus *Cryptaphelenchus* (Fuchs, 1937) seems to be monophyletic after available data (e.g. Pedram, 2019). The genus *Ektaphelenchoides* was erected by Baujard (1984), with *E. pini* (Massey, 1966; Baujard, 1984) as its type species. A compendium of the known species of the genus was given by Aliramaji et al. (2014). Since then, six further species were added to the genus (Esmaeili et al., 2014, 2018; Aliramaji et al., 2015, 2019a, 2019b; Golhasan et al., 2019), and it currently contains 18 species (http://www.organismnames.com). Following our recent studies on occurrence of aphelenchoidids (e.g. Aliramaji et al., 2018, 2020), and especially ektaphelenchid genera and species in Iran, a population of *Ektaphelenchoides* was recovered in natural forests in north of Iran. The spicules characters and the general morphology of the adults corroborated it belongs to the type species of the genus, *E. pini*. Thus, the aims of the present study were to characterize Iranian population of this species from Iran and discuss its morphological differences/similarities compared to available data and discuss its phylogenetic affinities. Furthermore, new taxonomic and morphological findings related with some other species of the genus are presented and discussed.

## Materials and methods

### Nematode extraction and morphological observations

Wood and bark samples (48 in total) were collected from natural forests of Golestan province in north of Iran, during 2019. The nematodes were extracted from the samples using the tray method (Whitehead and Hemming, 1965), handpicked under a Nikon stereomicroscope model SMZ1000, heat killed by adding boiling 4% formalin solution, transferred to anhydrous glycerin according to De Grisse (1969) and mounted on permanent slides. The slides were then studied and the nematodes were measured under a Nikon Eclipse E600 light microscope. Photographs were taken using an Olympus DP72 digital camera attached to an Olympus BX51 microscope equipped with differential interference contrast (DIC). The paratype specimens and the original descriptions of some previously described species of the genus were also studied in detail to amend their characterization or propose new taxonomic placements.

### DNA extraction, PCR, and sequencing

To prepare DNA samples, a single live nematode specimen of the recovered species was picked out, examined on a temporary slide and transferred to a small drop of TE buffer (10 mMTris-Cl, 0.5 mM EDTA, pH 9.0; Qiagen) on a clean slide and crushed using a cover slip. The suspension was collected by adding 20 μl TE buffer. The DNA sample was stored at −20°C until used as PCR template (two separate females were used for this purpose, and two DNA samples were prepared). The SSU rDNA was amplified using the forward primer F22 (5´-TCCAAGGAAGGCAGCAGGC-3´) (Dorris et al., 2002) and reverse primer 18S 1573R (5´-TACAAAGGGCAGGGACGTAAT-3´) (Mullin et al., 2005) (the several primers designed to amplify this fragment, e.g. two pairs designed by Holterman et al., 2006 did not yield on high-quality amplifications, or yield on no amplifications). The D2–D3 expansion segments of LSU rDNA were amplified using the forward D2A (5´-ACAAGTACCGTGAGGGAAAGT-3´) (Nunn, 1992) and reverse primer 1006R (5´-GTTCGATTAGTCTTTCGCCCCT-3´) (Holterman et al., 2008). The ITS1 region was amplified using the forward primer rDNA1 (5´-TTGATTACGTCCCTGCCCTTT-3´) and reverse primer rDNA1.58S (5´-ACGAGCCGAGTGATCCACCG-3´) (Subbotin et al., 2000). The polymerase chain reaction (PCR) cycles and sequencing of amplified fragments were according to Heydari et al. (2020). The newly obtained sequences of the species were deposited into GenBank database (accession number MT007525 for partial SSU and MT008125 for partial LSU rDAN D2-D3).

### Alignment and phylogenetic inference

For phylogenetic studies, two separate SSU and LSU datasets were prepared. Several available ektaphelenchid and seinurid species were included in both datasets. The SSU dataset included 50 sequences and the LSU dataset totaled 56 sequences (including newly generated sequences and sequences of the outgroup taxa), their accession numbers being given in the SSU and LSU phylogenetic trees. Each dataset was aligned using the QINS-i algorithm of online version of MAFFT version 7 (http://mafft.cbrc.jp/alignment/server/) (Katoh and Standley, 2013). The Gblocks program (version 0.91b) with all the three less stringent parameters, a server tool at the Castresana Lab (http://molevol.cmima.csic.es/castresana/Gblocks_server.html) was used for post-editing of the alignments, i.e., to eliminate poorly aligned regions or divergent positions. The Akaike-supported model, a general time reversible model, including among-site rate heterogeneity and estimates of invariant sites (GTR + G + I), was used in both phylogenies. The Bayesian analyses were performed using MrBayes v3.1.2 (Ronquist and Huelsenbeck, 2003) with a random starting tree, running the chains for 4 × 10^6^ generations. After discarding burn-in samples and evaluating convergence, the remaining samples were retained for further analyses. The Markov chain Monte Carlo (MCMC) method within a Bayesian framework was used to estimate the posterior probabilities of the phylogenetic trees (Larget and Simon, 1999) using the 50% majority rule. The convergence of model parameters and topology was assessed based on the average standard deviation of split frequencies and potential scale reduction factor values. Adequacy of the posterior sample size was evaluated using autocorrelation statistics, as implemented in Tracer v.1.6 (Rambaut and Drummond, 2009). The output files of the phylogenetic programs were visualized using Dendroscope V.3.2.8 (Huson and Scornavacca, 2012) and both SSU and LSU Bayesian trees were redrawn in CorelDRAW software version 17.

## Results

### Systematics

**Figure 1:**
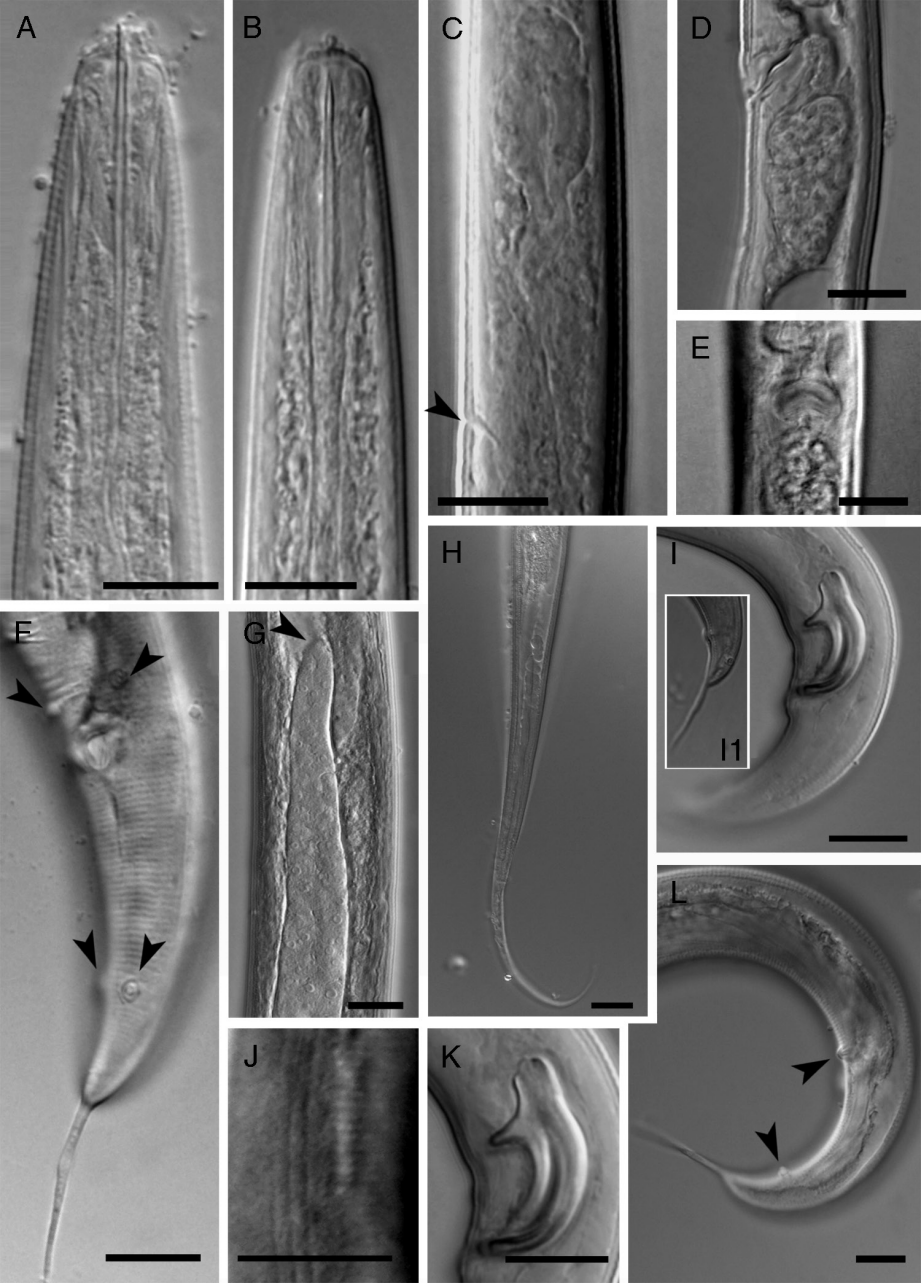
Iranian population of *Ektaphelenchoides pini* (Massey, 1966; Baujard, 1984). A: Anterior body region of male; B: Anterior body region of female; C: Metacorpus and the position of E pore (arrow) (male); D: Vulval region and PUS; E: Vulval slit in ventral view; F: Ventro-lateral view of male caudal region showing P2 (upper arrows) and P3 (lower arrows) papillae; G: Tip of ovary; H: Female posterior body region; I: Male cloacal region and spicule and tail tip (I1); J: Lateral lines; K: Spicule (close up), L: Male posterior body region showing P2 (upper arrow) and P3 (lower arrow) and single ventral P1 lacking. (Scale bars = 10 μm).

Iranian population of *Ektaphelenchoides pini* (Massey, 1966; Baujard, 1984) is shown in Figure 1.

### Measurements

Measurements of this population are given in Table 1.

**Table 1.** Morphometrics of *Ektaphelenchoides pini* (Massey, 1966; Baujard, 1984) from Golestan province, Iran, and comparison with data of the type population and the population from France.

	This study		Type population, studied by Baujard (1984)		French population (Baujard, 1984)	
Character	Females	Males	Female	Males	Females	Males
n	14	9	1	10	40	5
L	829.4 ± 70.2	611.8 ± 59.6	720	840	740	750
	(756–947)	(502–701)		(730–980)	(630–950)	(680–820)
a	37.4 ± 3.1	38.8 ± 3.2	33	32.5	33	33
	(30.0–37.7)	(33.8–39.6)		(28–38)	(31–36)	(31–36)
b	7.2 ± 0.8	6.7 ± 0.7	9	8	8	8
	(6.03–8.2)	4.3 ± 0.8		(7–9)	(7–11)	(7–9)
b´	4.7 ± 0.5	(3.3–5.8)	–	–	–	–
	(4–5.6)	13.9 ± 1.1				
c	–	(12.5–15.8)	–	14	–	14
		2.9 ± 0.2		(13–17)		(12.5–118.0)
c´	–	2.9 ± 0.2	–	2.9	–	3.1
		(2.7–3.1)		(2.6–3.0)		(2.2–3.6)
T or V	69.4 ± 2.0	90.6 ± 1.9	74	–	70	–
	(70.2–72.3)	(87.0–88.6)			(67–74)	
Cephalic region height	3.6 ± 0.4	3.0 ± 0.4	–	–	–	–
	(3.0-4.3)	(2.4-3.7)				
Cephalic region width	8.2 ± 0.5	7.4 ± 0.6	–	–	–	–
	(7.4–8.8)	(6.4–8.5)				
Stylet	20.9 ± 1.1	18.7 ± 1.0	23	24	23	23
	(20–23)	(17.3–19.8)		(21–26)	(20–26)	(22–24)
Max. body dim.	22.4 ± 2.2	15.8 ± 1.2	–	–	–	–
	(20–26)	(14–18)				
m	40.4 ± 5.0	44.4 ± 4.9	–	–	–	–
	(32.5–44.0)	(36.6–47.4)				
Body width at MB	17.1 ± 1.4	15.0 ± 1.2	–	–	–	–
	(16–20)	(13–17)				
E pore from anterior	97.5 ± 5.4	99.2 ± 8.3	104	119	105	
end	(92–106)	(91.2–108.1)		(101–132)	(94–123)	
Nerve ring from	100.5 ± 7.5	85.8 ± 14.2	–	–	–	–
anterior end	(87–97)	(71–110)				
Median bulb width	11.1 ± 1.4	9.9 ± 1.5	–	–	–	–
	(10–14)	(8–12)				
Median bulb length	20.7 ± 2.7	18.3 ± 2.3	–	–	–	–
	(17–25)	(16–22)				
Median bulb length/	1.9 ± 0.2	1.8± 0.1	–	–	–	–
diam. ratio	(1.5–2.2)	(1.7–2.0)				
Ovary/testis length	360.5 ± 34.7	197.8± 6.6	–	–	–	–
	(310–420)	(184–201)				
Hemizonid from	98 ± 3	104.4 ± 7.9	–	–	109	–
anterior end	(92–105)	(93–109)			(97–115)	
Vulval body diam.	22.3 ± 3.1	–	–	–	–	–
	(17–28)					
PUS length	27.6 ± 3.3	–	–	–	?	–
	(23–33)					
Anal/cloacal body	–	15.1 ± 1.5	–	–	–	–
diam.		(13–18)				
Tail	–	44.0 ± 3.1	–	–	–	53
		(40–49)				(38–61)
Spicules arc line	–	20.6 ± 1.0		25.5		24
length		(19–22)		(22–28)		(21–26)
Capitulum width	–	5.6 ± 0.5	–	–	–	–
		(5-6)				

**Note:** All measurements are in μm and in the form: mean ± s.d. (range).

### Female

Large nematodes. Body is slightly ventrally curved after fixation. Cuticle is finely annulated. Lateral fields have three incisures. Cephalic region is slightly set off from the body by a shallow depression. Stylet is well developed, its lumen is wide, and the conus is ca 38% of the total length with thick sclerotized walls, conophore, and knobs lacking. The protractor muscles are well developed, V-shaped, adjoining the stylet base to the cephalic framework. Procorpus is slender, wide, marked by a constriction at its junction with the median bulb; the latter is elongate ellipsoid, with anterior granular part occupying ca 30% of its length and post-central well-developed/sclerotized valves. Pharyngo-intestinal junction is immediately posterior to the base of median bulb. The dorsal lobe of pharyngeal glands forms a 101 to 148 μm long overlap. The intestine ends to a blind sac, rectum indistinct, and anus vestigial (in 11 females out of 14 examined individuals), which is presumably not functional. Nerve ring is located about one metacorpus length behind it. Hemizonid is immediately behind the E pore. The reproductive system is monodelphic-prodelphic, located on the right side of intestine, composing of an outstretched ovary with oocytes mostly at multiple rows behind germinal zone and tubular oviduct, axial spermatheca with small spheroid sperm in all examined females, the crustaformeria, uterus sometimes including sperm, anteriorly inclined 23 to 33 μm long vagina with sclerotized walls, vulva is a crescent-shaped slit without flap in ventral view and PUS ca 1.2 times the vulval body width long including sperm, apparently with no differentiation at the junction with uterus. Posterior body is elongated conoid, ending to a filiform tip.

### Male

Males are similar to females in anterior region morphology. Posterior body is strongly curved after fixation. The reproductive system is monarchic, located on the right side of intestine. Testis is outstretched, expanded anteriorly, not reflexed. Spermatocytes are in two to three rows in the germinal zone of the testis. Spicules are massive, wide, and their condylus is well-developed, marked by indentations at the apex, rostrum is developed with blunt tip, the lamina-calomus is complex and ventrally curved, and the distal tip of spicules is simple. Bursa and gubernaculum are absent. Two pairs of precloacal + caudal subventral papillae are present, the first pair (P2) is 5 to 6 μm anterior to cloacal opening, and the second pair (P3) is located at 30 to 41% of the tail, or 40 to 49 μm from the tail tip. The single precloacal papilla (P1) is lacking. Tail is dorsally convex, ventrally flat, with an 11 to 13 μm long spike ending.

### Related plant and locality

Recovered from the wood and bark samples of a dead broadleaf forest tree, collected in Golestan province, north of Iran, during October 2019, with GPS coordinates 36°41´36.9˝ N, 54°04´49.8˝E.

### Voucher specimens of Iranian population of *Ektaphelenchoides pini*

Three slides of the studied population of this species including eight females and four males were deposited in the USDA Nematode Collection, Beltsville, MD, USA (slides numbers: T-7401p to T-7403p).

### New observations on characteristics or taxonomic placements of some *Ektaphelenchoides* species

The detailed study of *Ektaphelenchoides maafiae* (Golhasan et al., 2019) revealed that it has close morphology with *E. tonekabonensis* (Aliramaji et al., 2019a) and has some minor morphometric differences, e.g. slightly longer stylet (13-15 vs 10.5-12.0 μm) in females and smaller c´ (3.4-4.5 vs 6.5-8.3) in males. However, according to light microphotographs (Fig. 2A, E in Golhasan et al., 2019), its stylet length is calculated 12 μm and the c´ value of male is calculated 5.85, and by identical 28S rDNA D2-D3 (few differences were observed that could be due to the quality of the reading of the sequence or its editing artefacts), and based on the Principle of Priority, is proposed as a junior synonym of *E. tonekabonensis*. The species lacks the cloacal pair of male caudal papillae (P2) (the observation of Aliramaji et al., 2019 is amended herein).

**Figure 2:**
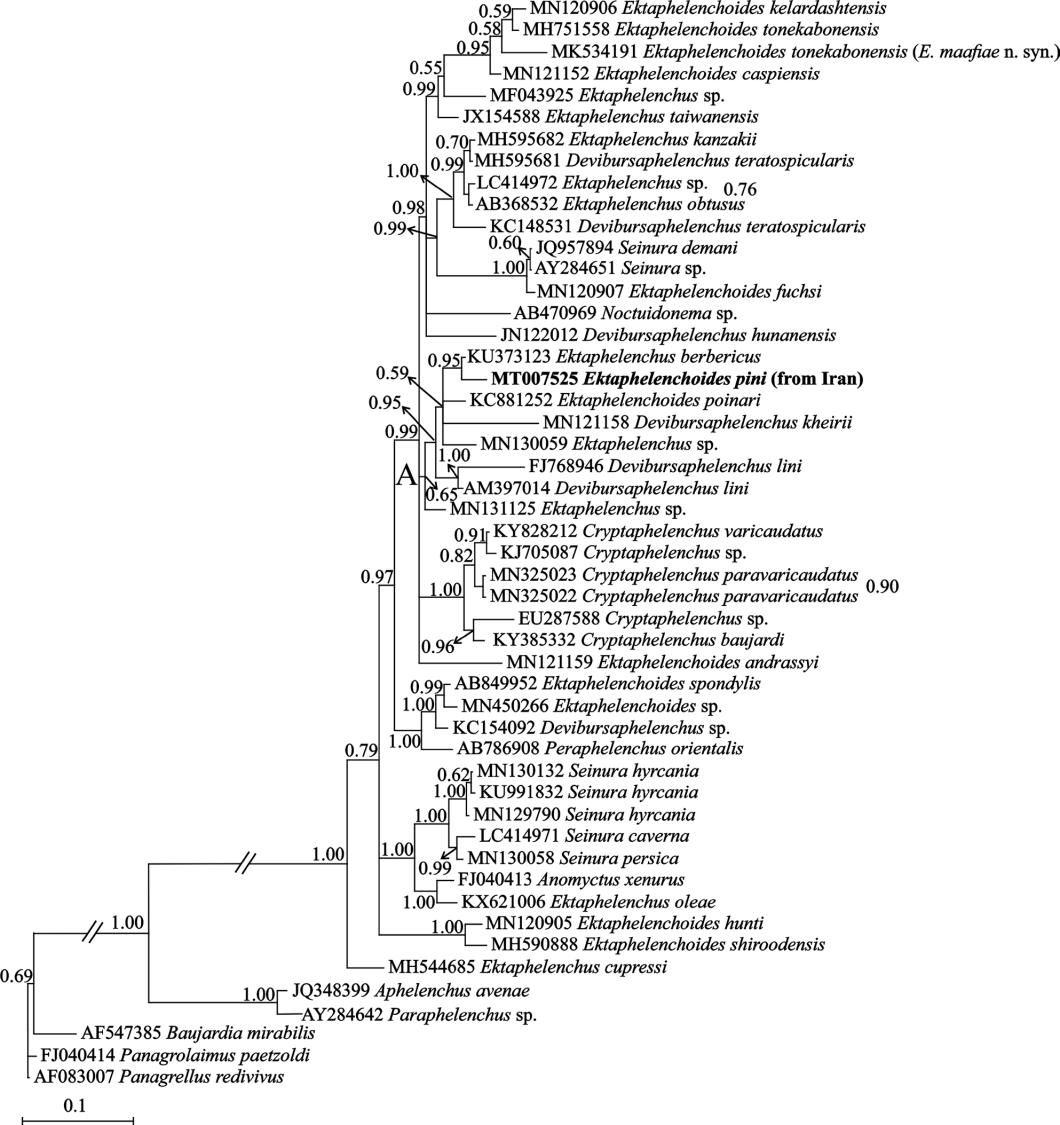
Bayesian 50% majority rule consensus tree inferred from analysis of the partial SSU rDNA sequence of Iranian population of *Ektaphelenchoides pini* (Massey, 1966; Baujard, 1984) from Golestan province under the GTR + G + I model. Bayesian posterior probability values more than 0.50 are given for appropriate clades. The new sequence is indicated in bold.

A review of the species of the genus revealed that *Ektaphelenchoides winteri* (Hooper, 1995) better fits the diagnostics of the genus *Ektaphelenchus* (Fuchs, 1937) by the posterior body end morphology of females and is proposed to be transferred to the latter genus.

The close study of the paratype male of *Ektaphelenchoides poinari* (Aliramaji et al., 2014) revealed that a slightly projecting structure is present slightly anterior to the cloacal opening. This could be the single precloacal P1 papilla, or the paired papillae, similar to those in *E. pini*. The last pair is situated at 14.5 μm distance to the tail tip. The arrangement and number of papilla for this species are amended herein.

### Molecular characterization and phylogenetic relationships

#### Partial SSU rDNA phylogeny

To determine the phylogenetic relationships of *Ektaphelenchoides pini* with other species, a newly obtained 1095 nt long partial sequence of SSU rDNA with accession number MT007525 was used. The BLAST search using this fragment revealed it is uniqueness and has 96.99% identity (32 mismatches and two indels in 1100 bp overlapping region) with *Ektaphelenchus berbericus* (Alvani et al., 2016) at maximum. Figure 2 represents the SSU phylogenetic tree inferred using the SSU data. In this tree, species of three genera *Ektaphelenchoides*, *Ektaphelenchus*, and *Devibursaphelenchus* (Kakuliya, 1967) have occupied distant placements, separate from each other. The newly generated sequence for the Iranian population of *Ektaphelenchoides pini* has fell into a highly supported clade (clade A) including species of three aforementioned ektaphelenchid genera. *Ektaphelenchus berbericus* (accession number KU373123) is the putative closest relative to *Ektaphelenchoides pini* in this tree.

#### D2 to D3 expansion segment of LSU rDNA phylogeny

**Figure 3:**
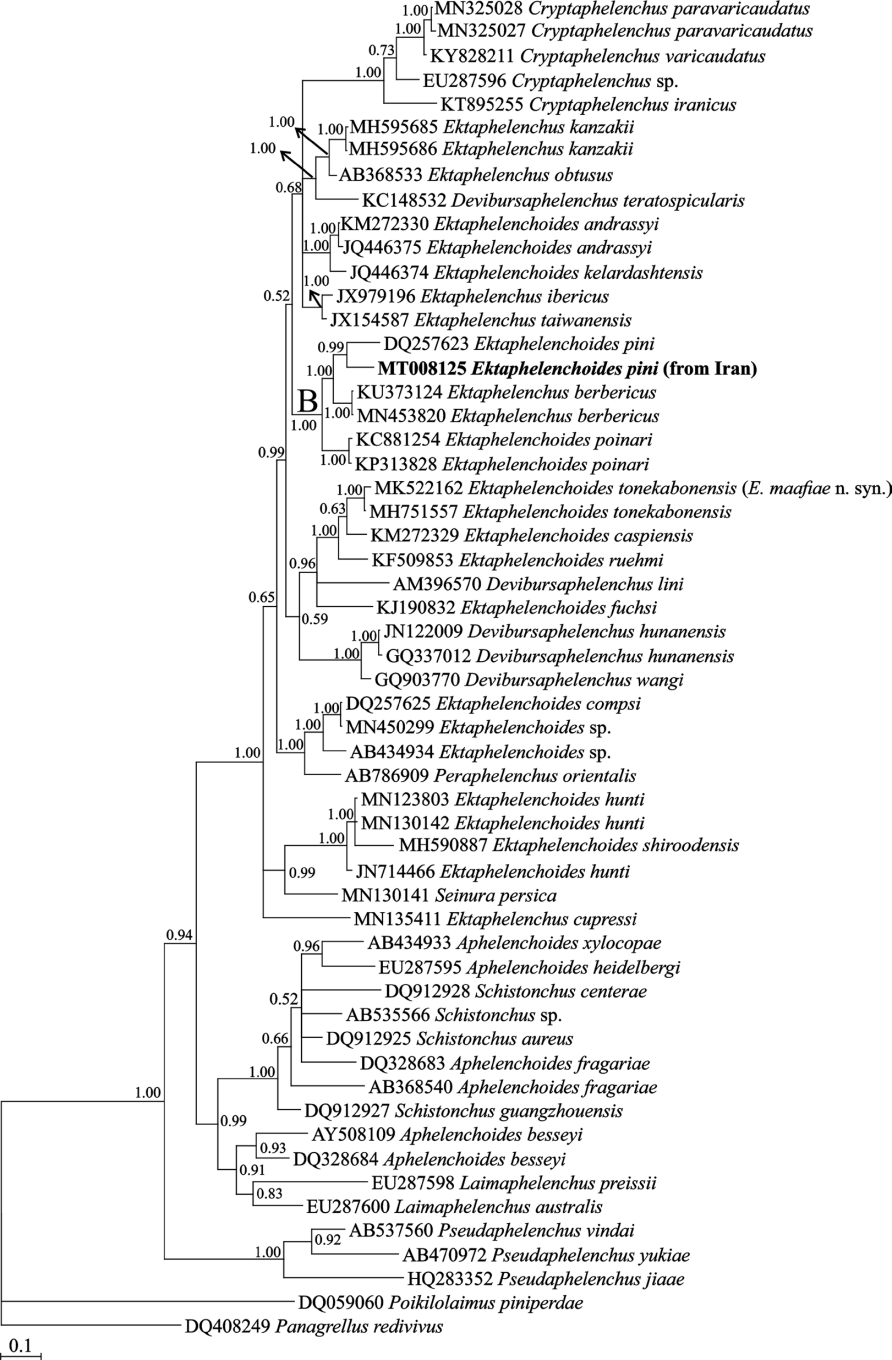
Bayesian 50% majority rule consensus tree inferred from analysis of the partial LSU rDNA sequence of Iranian population of *Ektaphelenchoides pini* (Massey, 1966; Baujard, 1984) from Golestan province under the GTR + G + I model. Bayesian posterior probability values more than 0.50 are given for appropriate clades. The new sequence is indicated in bold.

To reconstruct the LSU tree, a newly obtained 623 nt long sequence of D2 to D3 expansion segments of LSU rDNA with accession number MT008125 was used. The BLAST search using this fragment revealed it is unique and has 83.57% identity (99 mismatches and 13 indels) with a Chinese (?) isolate identified as *E. pini* and 93.66% identity (75 mismatches and 71indels) with *Ektaphelenchus berbericus* (KU373124). Figure 3 represents the phylogenetic tree inferred using the LSU dataset. In this tree, the newly generated sequence of Iranian population of *E. pini* is in sister relation with a Chinese (?) isolate (DQ257623) identified as *E. pini* with high (0.99) BPP. The clade of *E. pini* is inside the maximally supported clade B including four other sequences belonging to two genera *Ektaphelenchoides* and *Ektaphelenchus*, and *E. berbericus* (KU373124, MN453820) is its closest relative. Similar to SSU phylogeny, species of three genera *Ektaphelenchoides*, *Ektaphelenchus*, and *Devibursaphelenchus* have occupied distant placements, separate from each other.

## Discussion

The two genera *Ektaphelenchoides* and *Ektaphelenchus* are morphologically very similar, differentiated based upon the posterior body region shape of females, elongate, usually with filiform distal end in the former, and short, conical or cylindrical, in the latter (Kanzaki and Giblin-Davis, 2012). On the contrary, the assigning of the species to either of these genera has classically performed by several authors using the qualitative trait of posterior body end morphology of females, and currently there is no quantitative index to delimit them. During the present study, attempts were performed to delimit the species of both genera using the ratio of vulva to posterior body end distance, divided by the vulval body width, or, the distance from the blind end of rectum to distal body end, divided by the vulval body width. Although the calculated ratios could separate most traditionally classified species under each genus, but overlapping values were also observed (the data not shown). The details of this attempt as well as a proposal for re-defining these two genera are the subject of an independent study and will be presented elsewhere. A new synonymy, *Ektaphelenchoides maafiae* n. syn. a junior synonym of *E. tonekabonensis*, was proposed based on the Principle of Priority and the illustrated male caudal papillae for *E. tonekabonensis* (Aliramaji et al., 2019a) was amended. The same amendment was also performed for *E. poinari*. The detailed illustration of the male caudal papillae, especially in the case of the species with small body size, is usually difficult and need examination of fresh material in water in ventral view, or scanning electron microscopic data. In present study, it was found that *Ektaphelenchoides winteri* better fits the diagnostics of *Ektaphelenchus,* mainly by posterior body end morphology of females and was transferred to it as *Ektaphelenchus winteri* (Hooper, 1995) n. comb.

The type species of *Ektaphelenchoides*, *E. pini* was originally described from New Mexico (Massey, 1966). The species was originally briefly described. Baujard (1984) gave a redescription of the species in French and reported a second population from France. The species is mainly characterized by its spicules shape, having indentation(s) in condylus apex. The light microphotographs were not available for the both original and the second French population. The recovered population of species in present study made available an opportunity to characterize it in more detail and present its light microphotographs for the first time. In comparison to the type population (data from Baujard, 1984) and the French population reported by Baujard (1984), no remarkable morphological and morphometric differences were observed for the Iranian population. The PUS length in Iranian population is 1.2 to 1.3 times vulval body width. Its length was reported ‘1 body width in length’ by Massey (1966) and 0.3 to 0.95 times vulval body width by Baujard (1984).

In molecular phylogenetic analyses using partial SSU and LSU D2-D3 data, *Ektaphelenchoides berbericus* was the closest relative to *E. pini*. Besides morphological differences of two species (in brief: basically different shape of posterior body region of female in two species, i.e. elongate conoid, ending to a filiform tip in *E. pini* vs short conical, anteriorly located vulva (V = 69.4 (70.2-72.3) vs 80.2 (79.1-81.4)) and longer PUS (27 (23-33) vs 10.0 (7.0-13.5) μm) in *E. pini*), both species have remarkable differences in their SSU and LSU D2-D3 sequences as already discussed. As already known, the three ektaphelenchid genera including *Ektaphelenchoides*, *Ektaphelenchus*, and *Devibursaphelenchus* are not monophyletic based on these genomic markers (e.g. Pedram, 2019).

There are currently two available sequences submitted into the GenBank database under the name *E. pini*, a partial LSU sequence with the accession number DQ257623 that has remarkable differences with the newly generated LSU sequence for the Iranian population as already discussed, and a sequence with accession number DQ257620 for the ITS region. During present study, our efforts to sequence the ITS fragment were not successful. The observed differences between two Chinese (?) and Iranian LSU sequences are, however, difficult to interpret, as, the morphological data of the Chinese (?) population are not available. Such a remarkable difference between two LSU D2 to D3 sequences could either be due to the poor sequence quality of DQ257623, or misidentification of the studied population as *Ektaphelenchoides pini*. The latter case could neither be confirmed nor rejected by unavailable morphological data.

In the presently inferred SSU phylogeny, cladogenesis events were observed between several species, showing this marker could be used in molecular phylogenetic studies of ektaphelenchids.

### Remark

The repeated sequencing of D2 to D3 expansion segments using the second female revealed an identical sequence to the MT008125, and thus, only one sequence was used in LSU tree.
